# DeepTP: A Deep Learning Model for Thermophilic Protein Prediction

**DOI:** 10.3390/ijms24032217

**Published:** 2023-01-22

**Authors:** Jianjun Zhao, Wenying Yan, Yang Yang

**Affiliations:** 1 School of Computer Science and Technology, Soochow University, Suzhou 215006, China; 2 Collaborative Innovation Center of Novel Software Technology and Industrialization, Nanjing 210000, China; 3 Department of Bioinformatics, School of Biology and Basic Medical Sciences, Suzhou Medical College of Soochow University, Soochow University, Suzhou 215123, China; 4 Center for Systems Biology, Soochow University, Suzhou 215123, China; 5 Jiangsu Province Engineering Research Center of Precision Diagnostics and Therapeutics Development, Suzhou 215123, China

**Keywords:** thermophilic proteins, self-attention, convolutional neural network, bidirectional long short-term memory network, multiple-channel feature fusion

## Abstract

Thermophilic proteins have important value in the fields of biopharmaceuticals and enzyme engineering. Most existing thermophilic protein prediction models are based on traditional machine learning algorithms and do not fully utilize protein sequence information. To solve this problem, a deep learning model based on self-attention and multiple-channel feature fusion was proposed to predict thermophilic proteins, called DeepTP. First, a large new dataset consisting of 20,842 proteins was constructed. Second, a convolutional neural network and bidirectional long short-term memory network were used to extract the hidden features in protein sequences. Different weights were then assigned to features through self-attention, and finally, biological features were integrated to build a prediction model. In a performance comparison with existing methods, DeepTP had better performance and scalability in an independent balanced test set and validation set, with AUC values of 0.944 and 0.801, respectively. In the unbalanced test set, DeepTP had an average precision (AP) of 0.536. The tool is freely available.

## 1. Introduction

The thermal stability of proteins refers to the ability of proteins to maintain their unique chemical and spatial structures under high-temperature conditions. Protein engineering and biotechnology research largely rely on the thermal stability of proteins [1,2]. Thermophiles can produce thermophilic proteins that survive for a long time under high-temperature conditions without denaturation; some thermophilic proteins can even withstand 100 °C [3]. The high thermal stability of thermophilic proteins gives them outstanding advantages in industrial production. An extracellular isothermal cutinase (KERAK-29) was purified from thermophilic actinomycetes isolated from poultry compost, displaying the advantages of high heat resistance and fast catalytic reaction rate [4]. Thermostatic xylanases from thermophilic fungi have broad roles in food, feed, and the biotransformation of lignocellulose [5]. Therefore, the predictive study of thermophilic proteins is not only crucial for protein thermostability engineering, but it also has great value in practical fields such as industrial production.

Distinguishing thermophilic and mesophilic proteins through biological experiments is time-consuming, labor-intensive, and expensive. However, computational methods can quickly and accurately identify thermophilic and mesophilic proteins from a large amount of protein sequence information, which is an important topic in the field of protein thermal stability.

The thermal stability of proteins is closely related to biological characteristics such as amino acid composition, hydrogen bonds, salt bridges, and disulfide bonds. It was found that thermophilic proteins have more hydrophobic residues, charged residues, and aromatic residues than mesophilic proteins [6]. The different contents of various dipeptides and different types of hydrogen bonds also affect the thermal stability of proteins [7,8]. In some experiments, salt bridges, disulfide bonds, and other factors were found to improve thermal stability [9,10]. The biological characteristics of a protein are very important for the prediction of thermophilic proteins.

Computational methods for thermophilic protein prediction are mostly based on traditional machine learning methods. In earlier studies based on fewer datasets, researchers used the primary structure of protein sequences to obtain amino acid pairs, amino acid distribution, and basic features, and then used the logistic model tree algorithm, support vector machines (SVMs), and other classical algorithms to predict thermophilic proteins [7,11]. In recent years, researchers have expanded the thermophilic protein dataset. The amino acid composition and dipeptide propensity score were obtained, and then a prediction model called SCMTPP was constructed based on the scorecard method [12]. TMPpred [13] is a thermophilic protein predictor based on SVM, which shifts the focus to locating the important features affecting thermophilic proteins and analyzes an 188-dimensional feature set through an improved ANOVA feature selection method, locating the seven most important features. It was inferred that glycine, alanine, serine, and threonine are important factors affecting thermophilic proteins. SAPPHIRE [14] used an ensemble learning approach to predict thermophilic proteins, combining 12 feature encodings and 6 machine learning algorithms to train 72 baseline models. These studies have achieved certain results in predicting thermophilic proteins. However, they are all based on traditional machine learning. The datasets used are relatively small, and the features are relatively simple. This leaves a certain amount of room for improvement in accuracy and generalization.

The rapid development of deep learning technology has played a positive role in promoting the development of bioinformatics. Ahmed et al. [15] was the first to use deep learning technology to predict thermophilic proteins and proposed a thermophilic protein prediction model called iThermo. Combining the biological features of seven groups of protein sequences, a multilayer perceptron (MLP) was used to distinguish thermophilic proteins from mesophilic proteins.

Although the iThermo model uses a deep learning model, it only uses sequence-derived biological features and ignores information about the protein sequence itself. To extract the rich information contained in the protein sequence, this paper proposes a multi-channel thermophilic protein prediction model based on the self-attention mechanism, called DeepTP, which combines the hidden feature information of the protein sequence itself and sequence-derived biological features to predict thermophilic proteins. The method uses a convolutional neural network (CNN) to extract key local information from the protein sequence and then uses a bidirectional long short-term memory network (BiLSTM) to extract long-range dependent features. The key information of the protein sequence is then weighted by the self-attention mechanism. Finally, thermophilic protein prediction is achieved by MLP. Experimental results show that DeepTP performed better than other comparable methods on test and validation sets.

## 2. Results

### 2.1. Cross-Validation Performance of DeepTP

To build a model that could accurately identify thermophilic and mesophilic proteins, 797 features of six groups (amino acid composition [AAC], dipeptide composition [DPC], composition-transition distribution [CTD], quasi-sequence order descriptor [QSO], pseudo-amino acid composition [PAAC], and amphipathic pseudo-amino acid composition [APAAC]) of proteins were extracted. However, irrelevant and redundant features can affect model prediction performance. To overcome this problem, we combined the Light Gradient Boosted Machine (LightGBM) algorithm and recursive feature elimination algorithm based on cross-validation (RFECV) to select the features. The details of feature selection are described in Section 4.2. This reduced the number of features to 205. Subsequently, the performance of the model with 205 selected features was compared to that of the model with all features. The cross-validation performance of the models is shown in Table 1. The model using all features achieved an ACC of 0.872, MCC of 0.743, and AUC of 0.942, while the model using selected features achieved an ACC of 0.871, MCC of 0.742, and AUC of 0.943. In terms of performance, the model using all features was slightly better than the model using selected features. In terms of training time, the selected-features model needed 68,691 s, which was approximately 76% of the time needed by the all-features model. Therefore, the predictor using the selected features (205 features), named DeepTP, was chosen because a smaller number of features meant better coverage of the space of possible combinations and reduced the training time overhead.

### 2.2. Performance Comparison of DeepTP with Other Methods in the Independent Test Set and Validation Set

In recent years, existing tools for predicting thermophilic proteins have included TMPpred, SCMTPP, iThermo, and SAPPHIRE. We compared the performance of DeepTP with the above tools in an independent balanced test set, independent unbalanced test set, and validation set.

**Figure 1:**
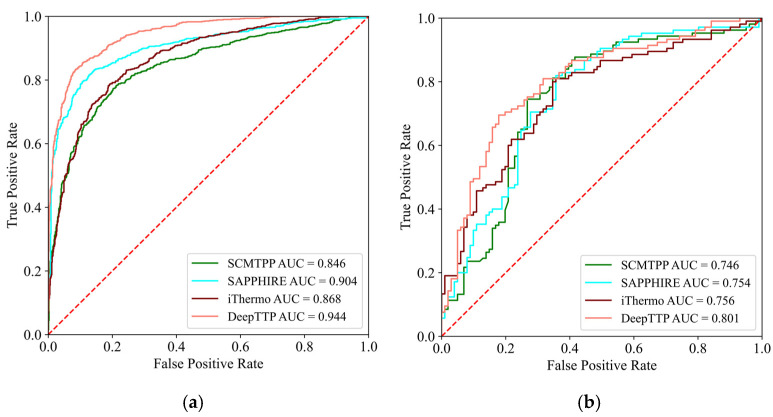
ROC curves on tools in (**a**) independent balanced test set. (**b**) validation set.

The performance of DeepTP and other tools in the balanced test set is shown in Table 2. The comprehensive performance of DeepTP was better, with an ACC of 0.873 and MCC of 0.746. Figure 1a shows the ROC curves of each model on the independent balanced test set, where the AUC of the DeepTP model was 0.944. The results returned on TMPpred’s prediction website did not contain predicted scores, and therefore, AUC values could not be calculated. This shows that DeepTP has higher accuracy and generalization capability than the other tools. SAPPHIRE is an ensemble learning model that achieved the second-best performance in the balanced test set. Table 2 shows that the PPV and SPE of SAPPHIRE were 0.911 and 0.930, respectively, but that the NPV and SEN were only 0.763 and 0.711, respectively. This shows that SAPPHIRE was more biased toward negative samples when predicting thermophilic proteins. However, the comprehensive performance of SAPPHIRE was lower than that of DeepTP, specifically, 5.2%, 8.9%, and 4.0% lower ACC, MCC, and AUC values than the DeepTP model, respectively. SCMTPP is a scorecard method (SCM)-based approach that uses the dipeptide composition of proteins with a 400-dimensional feature set. It also has the problem that its predictions are more biased toward negative samples.

TMPpred is a method based on traditional machine learning (support vector machines). It uses only seven features and its dataset is small, which may lead to its poor predictive performance on balanced tests. iThermo uses a deep learning (MLP) method, which showed worse performance than the DeepTP model, with 8.2%, 16.3%, and 7.6% lower ACC, MCC, and AUC values than the DeepTP model, respectively.

**Figure 2:**
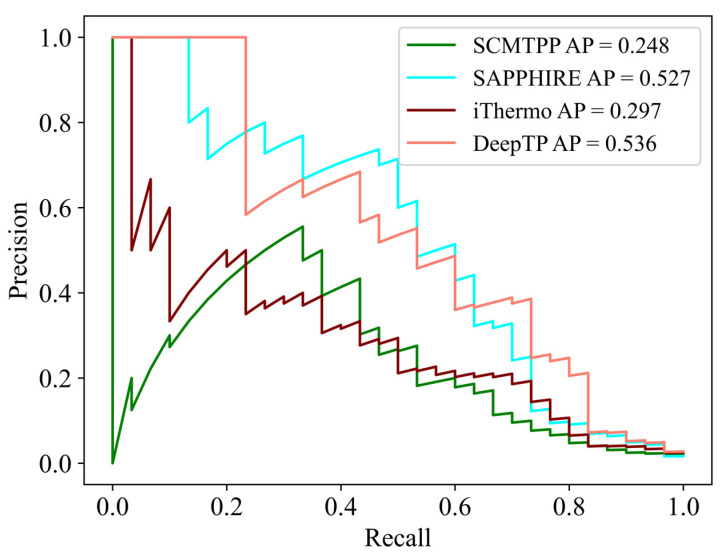
Precision-recall curves of tools in independent unbalanced test set.

In nature, there are far more mesophilic than thermophilic proteins. Therefore, in order to simulate this realistic situation, we next compared the performance of DeepTP with other tools in an unbalanced test set, which included 1800 mesophilic proteins and 30 thermophilic proteins. As shown in Figure 2 and Supplementary Table S1, SAPPHIRE had the highest performance, with PPV = 0.155, SPE = 0.933, ACC = 0.930, and MCC = 0.316, and DeepTP achieved better AP = 0.536, NPV = 0.997, and SEN = 0.833. The results demonstrated that DeepTP had better performance on the positive samples (thermophilic proteins), while SAPPHIRE had better performance on the negative samples (mesophilic proteins) in the unbalanced test set.

Overall, DeepTP performed better than other comparison tools in predicting thermophilic proteins in the above independent test sets. To further evaluate the performance of DeepTP publicly, the dataset provided by TMPpred on its website was obtained as a validation set, and one protein included in the training set was removed. A validation set containing 206 protein sequences was finally obtained. Five methods were compared on the validation set. The final performance of each method on the validation set is shown in Supplementary Table S2. Figure 1b shows the ROC curves of each model on the validation set, where the AUC of the DeepTP model was 0.801, which was highest on this almost balanced validation set. Altogether, DeepTP outperformed other comparable methods in both test and validation set. 

Since DeepTP only used the sequence information, another aspect of concern is the performance of DeepTP on homologous proteins, especially homologous mesophilic/thermophilic pairs. Hence, we also evaluated the performance on a homologous test set, which contained 100 thermophilic proteins and 100 mesophilic proteins with similarity higher than 40%. As shown in Supplementary Table S3 and Figure S2, DeepTP had the highest ACC (0.830), MCC (0.671), AUC (0.909), and AP (0.906) among the tools.

CNN and BiLSTM can be used to learn the features implicit in the protein sequence itself, after which the self-attention mechanism can be used to extract key features, fuse them with the biological features of the protein, and use the fused features to predict thermophilic proteins. This enables more important information to be obtained from the protein sequence and improves thermophilic protein prediction performance.

### 2.3. Algorithm Comparison

To verify the role of the various modules in the DeepTP model, three comparative experiments were designed to analyze the effects of these modules on model performance.

Comparison 1. Validate the effect of the two encoding modes on thermophilic protein prediction.

**Figure 3:**
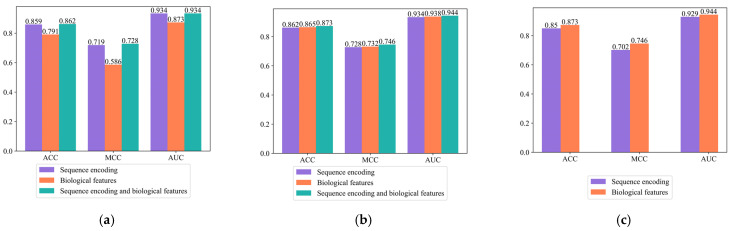
Comparative experiments performance. (**a**) The effect of different encoding modes on model performance. (**b**) The effect of using different features on model performance. (**c**) The effect of self-attention on model performance.

Three models were constructed using only the amino acid composition encoding mode, using only the amino acid physicochemical property encoding mode, and using both encoding modes. As can be seen from Figure 3a and Supplementary Table S4, when the amino acid composition encoding mode or the amino acid physicochemical property encoding mode was used alone, the ACC values of the model were 0.859 and 0.791, and the MCC values were 0.719 and 0.586, respectively. When the two encoding modes were combined, the ACC of the model was 0.862 and the MCC was 0.728. Combining the two encoding modes was more advantageous than a single encoding, indicating that the combined encoding method brought about a certain improvement in thermophilic protein prediction performance.

Comparison 2. Validate the effect of the fused biological features on thermophilic protein prediction.

Contrastive experiments using sequence encoding, biological features, and sequence encoding of fused biological features were designed. As shown in Figure 3b and Supplementary Table S5, when only sequence encoding was used, the ACC of the model was 0.862 and the MCC was 0.728. When only biological features were used, the ACC of the model was 0.865 and the MCC was 0.732. After the fusion of sequence encoding and biological features, the ACC of the model improved to 0.873 and the MCC improved to 0.746, indicating that the fused biological features predicted thermophilic proteins more effectively.

Comparison 3. Validate the effect of adding a self-attention mechanism on thermophilic protein prediction.

Comparative experiments with and without the self-attention mechanism were designed. As can be seen from Figure 3c and Supplementary Table S6, the ACC of the model with the self-attention mechanism improved by 2.3% compared to that of the model without the self-attention mechanism, and the MCC improved by 4.4%. Using the self-attention mechanism can better extract key information on protein sequences, thereby improving model performance.

### 2.4. DeepTP Web Application

DeepTP is freely available as a web application at http://www.YangLab-MI.org.cn/DeepTP (accessed on 20 January 2023). The program uses as input protein sequence(s). DeepTP provides a complete report, which is sent to the user by email when ready. The website contains datasets used for training and testing, as well as the results for the predictions of three proteomes.

## 3. Discussion

Biological experiments are a time-consuming and labor-intensive way to determine thermophilic proteins, and therefore, computational tools are needed for this task. Thermophilic proteins have high thermal stability and play an important role in industrial production, life sciences manufacturing, and other fields. The application of deep learning in the field of bioinformatics is becoming more extensive. With the development of sequencing technology, large amounts of protein sequence information are being generated, meaning that comprehensive analysis of thermophilic proteins can be performed based on their sequences. In this study, a new predictor based on protein sequences and deep learning was developed, called DeepTP.

There is no large-scale public benchmark dataset for thermophilic protein prediction. Therefore, the authors constructed a reliable large-scale benchmark dataset, calculated six sets of biological features, and used RFECV to filter out the optimal feature subset.

The protein sequence itself contains rich information. The protein sequence was encoded in two ways: through encoding of amino acid composition and amino acid physicochemical properties. Then, the encoded sequence was extracted by CNN, BiLSTM, and the self-attention mechanism, and finally, the sequence features extracted by deep learning were fused with the biological features to construct a thermophilic protein predictor.

Due to the lack of benchmark datasets, two independent test sets were constructed, and the dataset provided by TMPpred was obtained as a validation set. DeepTP was compared with TMPpred (accessed on 13 January 2023), SCMTPP (accessed on 13 January 2023), iThermo (accessed on 13 January 2023), and SAPPHIRE (accessed on 20 January 2023) in the test and validation sets, achieving the highest AUC values among these tools, with 0.944 in the balanced test set, 0.940 in the unbalanced test set, and 0.801 in the validation set. In the balanced test set, the ACC of DeepTP was at least 5.2% higher and the MCC was at least 8.9% higher.

In the unbalanced test set and validation set, DeepTP did not achieve the highest ACC or MCC, but had better AP and AUC. SAPPHIRE had the highest performance on the unbalanced test set in PPV, SPE, ACC, and MCC. SAPPHIRE is a stacking-based ensemble learning framework, which employs various feature encoding schemes and integrates an optimal combination of baseline models. The comprehensive feature exploration provides sufficient information from multiple perspectives, and the baseline model integration and optimization decreases the generalization error rate of single machine learning based classifiers. DeepTP adopted a deep learning strategy based on self-attention and multiple channel feature fusion. The better performance of SAPPHIRE than DeepTP is primarily owing to the comprehensive exploration of different feature encodings to obtain sufficient information and careful analysis of the relationship between prediction results and each feature. Indeed, interpretability is one of major technical obstacles in the implementation of deep learning. In future studies, more biological features with comprehensive feature optimization might be integrated into our approach to enhance the prediction performance.

Three experiments were constructed to analyze the model. The results showed that combining amino acid composition encoding and amino acid physicochemical property encoding more fully expressed the protein sequence. Use of the self-attention mechanism better captured key information about the amino acid residues. The fusion of biological features with sequence features acquired by deep learning technology provided superior prediction performance for thermophilic proteins.

In conclusion, a multi-channel thermophilic protein prediction model has been proposed based on a self-attention mechanism. The approach uses CNN and BiLSTM to learn the hidden features of the protein sequence itself and then uses the self-attention mechanism to weight the obtained features, extract the corresponding key features, and fuse them with the biological features of the protein sequence to build a thermophilic protein prediction model. Future work will involve attempts to incorporate more effective biological features and new model architectures to reconstruct the model and improve its performance. Efforts will also be made to predict thermophilic proteins using semi-supervised and unsupervised methods.

The tool is freely available and allows the submission of sequence information in different formats.

## 4. Materials and Methods

### 4.1. Datasets

**Figure 4:**
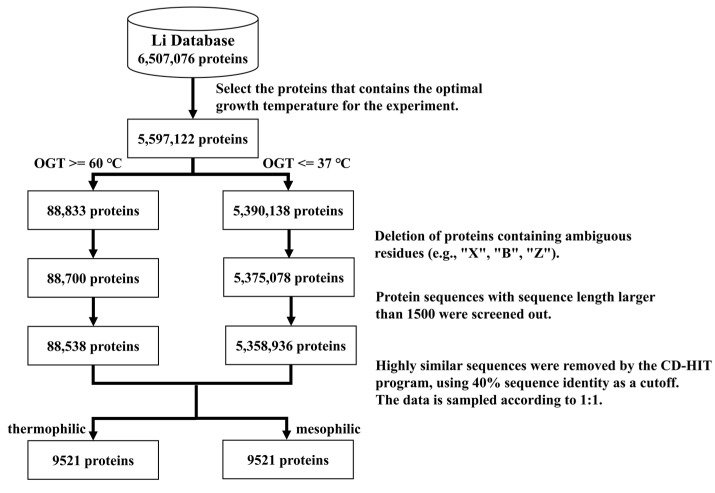
The procedure of dataset construction.

1. There are no large-scale public datasets of thermophilic proteins for the proposed computational methods, all of which use small sample data. Li et al. [16] constructed a database containing experimental optimal protein growth temperatures and predicted optimal temperatures; their experimental data were used in this study. The following steps were taken to ensure the quality of the dataset (Figure 4):
  1. The proteins with known optimal growth temperatures from the database of Li et al. were kept, resulting in 5,597,122 proteins.
  2. Thermophilic proteins were defined as proteins with 60 °C as their lowest optimal growth temperature [11], while 37 °C was chosen as the highest optimal growth temperature for mesophilic proteins. The 60 °C cutoff was for hyperthermophiles rather than average thermophiles.
2. All protein sequences were extracted from Uniprot [17]. Sequences that contained other protein fragments or had more than 1500 residues were excluded.
3. Highly similar sequences were removed using the CD-HIT [18] program, applying 40% sequence identity as a cutoff.
4. The number of mesophilic proteins in the dataset obtained by the above steps was much greater than the number of thermophilic proteins. To avoid the influence of data imbalance, the data were under-sampled by randomly deleting some mesophilic proteins. The numbers of thermophilic proteins and mesophilic proteins were thus made the same.
5. The final training set included 8704 thermophilic proteins and 8704 mesophilic proteins. The balanced test set consisted of 817 thermophilic proteins and 817 mesophilic proteins.
6. In nature, there are far more mesophilic than thermophilic proteins. Therefore, in order to simulate this realistic situation, we also constructed an independent unbalanced test set, which included 30 thermophilic proteins and 1800 mesophilic proteins to keep the same proportion of positive and negative samples as in the original Li et al. database. Thirty thermophilic proteins were random selected from the test set and 1800 mesophilic proteins were random selected from all mesophilic proteins, excluded the proteins in the training set.

The validation set came from TMPpred [13] and one protein included in the training set was removed. The final TMPpred validation set contained 101 mesophilic and 105 thermophilic proteins.

### 4.2. Features

To build a model that could accurately identify thermophilic and mesophilic proteins, the features of six groups of proteins were extracted using the protr [19] program, namely amino acid composition (AAC), dipeptide composition (DPC), composition-transition distribution (CTD), quasi-sequence order descriptor (QSO), pseudo-amino acid composition (PAAC), and amphipathic pseudo-amino acid composition (APAAC). Finally, 797 features were obtained. Table 3 lists the number of features for each class. Details of the features can be found in the Supplementary Features Description.

### 4.3. Feature Selection

Irrelevant and redundant features can affect model prediction performance. If the feature dimension is too large, the model will have difficulty converging during training. To reduce the influence of irrelevant and redundant features on the model and reduce the training time, a feature-selection method was used to remove irrelevant and redundant features. With reference to the feature-selection method used by ProTstab (accessed on 13 January 2023) [20,21], the LightGBM algorithm was adopted, and the recursive feature elimination algorithm based on cross-validation (RFECV) was chosen for feature selection. Recursive feature elimination (RFE) [22] requires specifying the number of features required, but usually it is not possible to determine how many features are valid. Cross-validation and RFE algorithms were used together to score different feature subsets and select the optimal subset, which was an efficient feature-selection scheme. Finally, 205 biological features were selected in addition to the feature representations obtained from deep learning to train the model.

### 4.4. Model

**Figure 5:**
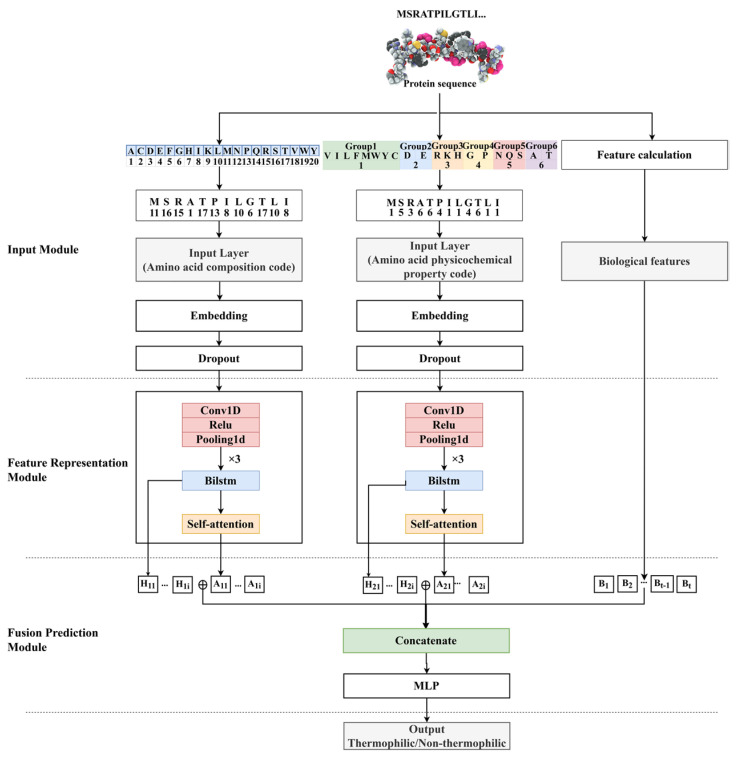
DeepTP workflow.

DeepTP is a multi-channel feature fusion prediction model based on the self-attention mechanism. The prediction procedure of the model is shown in Figure 5. The detailed forecasting process is as follows: (a) the input was the three vectors of the protein sequence after amino acid composition encoding, amino acid physicochemical property encoding, and normalization of biological features. The vectors processed by the two encoding modes performed the subsequent operations concurrently. (b) The vectors encoded by the protein sequences are mapped to dense vectors through the embedding layers. To avoid overfitting, part of the information was lost through the dropout layer. (c) The vectors were sent to CNN to extract key local features in the sequences. (d) The feature information hidden deep in the sequences was obtained through the BiLSTM layer, and the relationship between long-range dependencies was explored. The corresponding hidden units were then extracted. (e) The attention mechanism was used to weight key information in the sequences, assigning more attention to important information and less attention to unimportant information. (f) The long-range dependencies were extracted by the BiLSTM layer, the key information was extracted by the attention layer, and the biological features were integrated. The fused features were sent to the multi-layer perceptron for nonlinear transformation, and the sigmoid function was used to complete the final prediction.

#### 4.4.1. Input Module

The input of the DeepTP model included amino acid composition encoding, amino acid physicochemical property encoding, and protein sequence-based biological features, as shown in Figure 5. 

**Amino acid composition encoding**

The protein sequence was encoded according to the abbreviated alphabetical order of the amino acid residues, with each amino acid corresponding to a specific real number.

**Amino acid physicochemical property encoding**

There is a close relationship between the physicochemical properties of amino acids and thermophilic proteins. The amino acids were divided into six groups according to their physical and chemical properties [23]: hydrophobic (V, I, L, F, M, W, Y, C), negatively charged (D, E), positively charged (R, K, H), conformation (G, P), polarity (N, Q, S), and other properties (A, T). The amino acids were encoded in sequence according to the class. Encoding details are shown in Supplementary Table S8.

**Protein sequence-based biological features**

The biological features are detailed in Section 2.2 and Section 2.3; the final number of biometrics used was 205.

The two vectors encoded by amino acid composition and amino acid physicochemical properties were input into the embedding layer and mapped to dense vectors. Injecting noise (such as dropouts) into hidden units can effectively prevent the model from overfitting. Therefore, a dropout layer was added after the embedding layer to temporarily drop some of the neural network units from the network.

#### 4.4.2. Feature Representation Module

As shown in Figure 5, after the input protein sequence was processed by the input module, it was input into the feature representation module to extract the internal information of the sequence. The feature representation module consisted of three parts: the CNN module, the BiLSTM module, and the self-attention mechanism module.

**CNN module**

A CNN [24] can effectively capture key local features, thus CNN was used to analyze protein sequences. The convolution module set up three convolutional network layers. Each convolutional layer used local connections and weight sharing to perform convolution operations on the data to obtain key local information. The first convolutional layer had 128 filters, and the second and third convolutional layers had 64 filters, each with a sliding step of 1. After a series of convolution operations, feature maps with higher dimensions $c_{1}$ and $c_{2}$ were created.

Using a pooling layer can effectively reduce the size of the parameter matrix, thereby reducing the number of parameters in the model. Therefore, adding pooling layers can improve computational efficiency and avoid overfitting. Therefore, a max pooling operation was performed in the pooling layer to obtain the outputs $c_{1}^{\prime }$ and $c_{2}^{\prime }$.

**BiLSTM module**

Prediction of thermophilic proteins uses information from the entire sequence, and prediction model performance may be affected by dependencies that exist between sequence contexts. Therefore, the BiLSTM [25] algorithm was used to obtain further dependency information between protein sequence contexts. The structure of BiLSTM is shown in Supplementary Figure S3.

The forward layer of BiLSTM performed forward calculation from time 1 to *t* and obtained the output of the forward hidden layer at each time. From time *t* to 1, the backward layer performed reverse calculations to obtain the output of the backward hidden layer at each time. On this basis, the outputs of the forward layer and the backward layer at each moment were combined to obtain the final output result:

(1) $$C_{f}=f\left(w_{1}x_{t}+w_{2}C_{f-1}\right),$$

(2) $$C_{b}=f^{\prime }\left(w_{3}x_{t}+w_{5}C_{b-1}\right),$$

(3) $$H_{m}=g\left(w_{4}C_{f}+w_{6}C_{b}\right),$$

where *t* represents time; *x* represents the input; $w_{i}$ is the weight; $C_{f}$ is the output of the forward layer; $C_{b}$ is the output of the backward layer; f() and $f^{\prime }()$ calculate the outputs of the forward and backward layers, respectively; and g() combines and sums the outputs of the forward and backward layers. Finally, the output ($H_{m}$) of the BiLSTM layer was generated.

The output of the convolution module was input into the BiLSTM layer, and two 128-dimensional feature vectors $H_{1}$ and $H_{2}$ were finally obtained after training.

**Self-attention mechanism module**

The introduction of the attention mechanism can help the model to assign different weight values to each part of the input, thereby extracting key information and enabling the model to make more accurate decisions. Attention mechanisms are widely used in various fields.

The self-attention mechanism [26] efficiently processes a given level of information in parallel. Based on the fully extracted protein sequence feature information obtained through the CNN and BiLSTM modules, the self-attention mechanism was used for optimization so that the model could pay more attention to key information in the protein sequence, thereby enhancing the module’s ability to extract key features. The computation of the self-attention mechanism was carried out as follows.

The input word vector matrix was first mapped into three spaces to obtain three vectors *Q*, *K*, and *V*. The expressions are:

(4) $$Q=EW_{i}^{Q},$$

(5) $$K=EW_{i}^{K},$$

(6) $$V=EW_{i}^{V},$$

where *Q*, *K*, and *V* represent the matrices composed of query, key, and value vectors, respectively, and $W_{i}^{Q}$, $W_{i}^{K}$, and $W_{i}^{V}$ are the parameter matrices of the *i-th* linear mapping.

Point multiplication was used to calculate the similarity between *K* and *Q*, after which the softmax() function was used to normalize the attention weights to obtain the probability distribution according to the following expression:

(7) A=softmax(K·Q).

Finally, the weights *A* and *V* were weighted and summed to obtain the attention, for which the expression is:

(8) Attention(Q,K,V)=V·A.

After the hidden features of the extracted protein sequences were processed by the self-attention mechanism module, more attention was allocated to important features and less attention to unimportant features, and finally, outputs $A_{1}$ and $A_{2}$ were obtained.

#### 4.4.3. Prediction Fusion Module

The dimension of the biological features also affects the predictive performance of the model. If the dimension of the biological feature is too large, the feature dimension obtained after fusion with the output feature of deep learning will be too large, which will increase the complexity of model prediction. Therefore, biological features B were used after feature selection and standardized processing.

The feature vectors of the BiLSTM layer, the output vectors of the self-attention mechanism layer, and the biological feature vector were fused as the input to the next layer.

The result after feature fusion was input into the MLP. The MLP is connected through three fully connected layers, and each layer of nodes has a ReLu activation function. At the same time, to avoid overfitting, three dropout layers were added between the fully connected layers. Finally, the sigmoid activation function turned the output into a value in the range (0, 1).

The specific experimental parameter settings can be found in Supplementary Table S7.

### 4.5. Evaluation Metrics

Thermophilic protein prediction is a binary classification problem. Seven indicators are used to comprehensively evaluate the prediction model: positive predictive value (PPV), negative predictive value (NPV), sensitivity, specificity (SPE), accuracy (ACC), Matthews correlation coefficient (MCC), the area under the receiver operating characteristic curve (AUC), and the average precision (AP). These metrics are calculated as follows:

(9) $$\mathrm{PPV}\;\left(\mathrm{Precision}\right)=\frac{TP}{TP+FP},$$

(10) $$\mathrm{NPV}=\frac{TN}{TN+FN},$$

(11) $$\mathrm{SEN}\;\left(\mathrm{Recall}\right)=\frac{TP}{TP+FN},$$

(12) $$\mathrm{SPE}=\frac{TN}{TN+FP},$$

(13) $$\mathrm{ACC}=\frac{TP+TN}{TP+TN+FP+FN},$$

(14) $$\mathrm{MCC}=\frac{TP\times TN-FP\times FN}{\sqrt{\left(TP+FN\right)\times \left(TP+FP\right)\times \left(TN+FN\right)\times \left(TN+FP\right)}},$$

(15) $$\mathrm{AUC}=\frac{\sum_{i\in positives}rank_{i}-\frac{M\left(1+M\right)}{2}}{M\times N},$$

(16) $$\mathrm{AP}\;=\underset{n}{\sum }\left({Recall}_{n}-{Recall}_{n-1}\right)\times {Precision}_{n}$$

where *TP* represents the number of correctly predicted thermophilic proteins, *FP* represents the number of incorrectly predicted thermophilic proteins, *FN* represents the number of incorrectly predicted mesophilic proteins, *TN* represents the number of correctly predicted mesophilic proteins, *positives* represents the set of positive samples, *M* represents the number of positive samples, and *N* represents the number of negative samples. $Recall_{n}$ and $Precision_{n}$ represent the precision and recall at the *nth* threshold, respectively. The predicted scores of the samples are then arranged in ascending order, with the lowest score being *rank1* and so on to obtain $rank_{i}$.

For the balanced datasets, the AUC curve was used to evaluate the performance, and the precision-recall curve was preferred for the unbalanced set.

## Figures and Tables

**Table 1:** 10-fold cross-validation performance in training set.

Evaluation Indicators	With All 797 Features	With 205 Selected Features (DeepTP)
PPV	0.876 ± 0.012	0.870 ± 0.016
NPV	0.868 ± 0.011	0.873 ± 0.012
SEN	0.866 ± 0.013	0.873 ± 0.010
SPE	0.878 ± 0.009	0.869 ± 0.014
ACC	0.872 ± 0.007	0.871 ± 0.007
MCC	0.743 ± 0.014	0.742 ± 0.013
AUC	0.942 ± 0.004	0.943 ± 0.004
TIME (s)	90,042	68,691

The number is mean ± standard deviation.

**Table 2:** Performance comparison of different methods in the independent balanced test set.

Evaluation	TMPpred	SCMTPP	iThermo	SAPPHIRE	DeepTP
PPV	0.731	0.864	0.817	0.911	0.887
NPV	0.689	0.704	0.768	0.763	0.860
SEN	0.659	0.621	0.749	0.711	0.854
SPE	0.758	0.902	0.832	0.930	0.891
ACC	0.708	0.761	0.791	0.821	0.873
MCC	0.418	0.545	0.583	0.657	0.746
AUC	-	0.846	0.868	0.904	0.944
AP	-	0.857	0.867	0.916	0.946

**Table 3:** Feature information.

Feature Type	Description	Dimension
AAC	Frequency of 20 amino acids	20
DPC	Frequency of 400 dipeptides	400
CTD	Composition, transition, and distribution	147
QSO	Distance matrix between 20 amino acids	100
PAAC	Pseudo-Amino Acid Composition	50
APAAC	Amphiphilic Pseudo-Amino Acid Composition	80
Total	-	797

## Data Availability

The datasets and source codes are available at https://github.com/ZhaoDove/DeepTP_predictor.

## Supplementary Materials

The following supporting information can be downloaded at: https://www.mdpi.com/article/10.3390/ijms24032217/s1. References [27,28,29,30] are cited in the supplementary materials.
