# Supplementary material for: DeepTP: A Deep Learning Model for Thermophilic Protein Prediction
Source: Int J Mol Sci. 2023 Jan 22;24(3):2217. doi: 10.3390/ijms24032217 (PMC9917291; doi:10.3390/ijms24032217)
Supplement: Supplementary file 1 [file ijms-24-02217-s001.zip › ijms-2122047-supplementary.pdf]

DeepTP: A deep learning model for thermophilic protein prediction

JianjunZhao[1,2], Wenying Yan[3,4,5], Yang Yang[1,2]

[1]School of Computer Science and Technology, Soochow University, Suzhou, 215006, China

[2]Collaborative Innovation Center of Novel Software Technology and Industrialization, Nanjing, 210000, China

[3] Department of Bioinformatics, School of Biology and Basic Medical Sciences, Suzhou Medical College of Soochow University, Soochow University, Suzhou 215123, China

[4] Center for Systems Biology, Soochow University, Suzhou 215123, China

[5] Jiangsu Province Engineering Research Center of Precision Diagnostics and Therapeutics Development, Suzhou 215123, China

Supplementary Materials

1. Precision-recall curves on tools in balanced test set and validation set

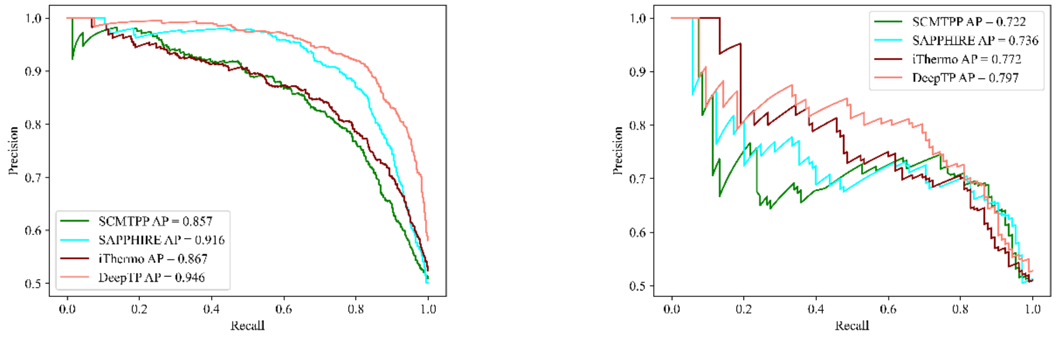

(a) (b)

Supplementary Figure S1. Precision-recall curves on tools in (a) balanced test set and (b) validation

set.

2. Performance table for independent unbalanced test

Supplementary Table S1. Performance comparison of different methods in the independent unbalanced test set

Evaluation	TMPpred	SCMTPP	iThermo	SAPPHIRE	DeepTP
TP	22	22	24	22	25
TN	1304	1592	1465	1680	1597
FP	496	208	335	120	203
FN	8	8	6	8	5
PPV	0.042	0.098	0.067	0.155	0.110
NPV	0.994	0.995	0.996	0.995	0.997
SEN	0.733	0.733	0.800	0.733	0.833
SPE	0.724	0.884	0.814	0.933	0.887
ACC	0.725	0.882	0.814	0.930	0.886
MCC	0.129	0.237	0.196	0.316	0.277
AP	-	0.248	0.297	0.527	0.536

3. Performance table for validation set

Supplementary Table S2. Performance comparison of different methods in the validation set

Evaluation	TMPpred	SCMTPP	iThermo	SAPPHIRE	DeepTP
PPV	0.688	0.736	0.720	0.696	0.833
NPV	0.684	0.664	0.664	0.584	0.605
SEN	0.708	0.632	0.638	0.457	0.429
SPE	0.663	0.762	0.743	0.792	0.911
ACC	0.686	0.696	0.689	0.621	0.665
MCC	0.371	0.397	0.382	0.264	0.386
AUC	-	0.746	0.756	0.754	0.801
AP	-	0.722	0.772	0.736	0.797

4. Performance comparison for homologous mesophilic/thermophilic pairs

Supplementary Table S3. Performance comparison of different methods in homology test set

Evaluation	TMPpred	SCMTPP	iThermo	SAPPHIRE	DeepTP
TP	69	73	78	79	92
TN	63	71	70	78	74
FP	36	29	30	22	26
FN	31	27	22	21	8
PPV	0.657	0.716	0.722	**0.782**	0.780
NPV	0.670	0.724	0.761	0.788	**0.902**
SEN	0.690	0.730	0.780	0.790	**0.920**
SPE	0.636	0.710	0.700	**0.790**	0.740
ACC	0.663	0.720	0.740	0.785	**0.830**
MCC	0.327	0.440	0.482	0.570	**0.671**
AUC	-	0.808	0.834	0.871	**0.909**
AP	-	0.792	0.836	0.862	**0.906**

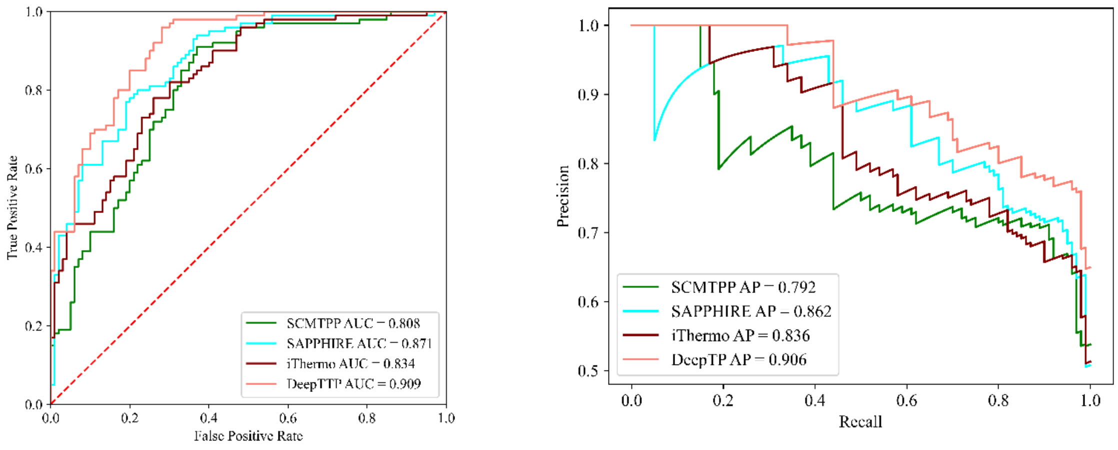

(a) (b)

Supplementary Figure S2. (a) AUC curves and (b) precision-recall curves on tools in homologous test set

5. Performance table for different experiments

Three experiments have been added to verify the role of different modules of DeepTP. Supplementary Tables S4, S5, and S6 are the performance of the verification experiments.

Supplementary Table S4. Performance of different encodings

Evaluation	Amino acid composition encoding	Amino acid physicochemical properties encoding	Using both encodings
PPV	0.871	0.831	**0.899**
NPV	**0.848**	0.759	0.832
SEN	**0.843**	0.729	0.816
SPE	0.875	0.852	**0.908**
ACC	0.859	0.791	**0.862**
MCC	0.719	0.586	**0.728**
AUC	**0.934**	0.873	**0.934**

Supplementary Table S5. Model performance using different features

Evaluation	Sequence encoding	Biological features	Sequence encoding and biological features
PPV	**0.899**	0.884	0.887
NPV	0.832	0.848	**0.860**
SEN	0.816	0.841	**0.854**
SPE	**0.908**	0.890	0.891
ACC	0.862	0.865	**0.873**
MCC	0.728	0.732	**0.746**
AUC	0.934	0.938	**0.944**

Supplementary Table S6. The effect of the self-attention on model performance

Evaluation	Without self-attention	With self-attention
PPV	0.878	**0.887**
NPV	0.826	**0.860**
SEN	0.813	**0.854**
SPE	0.887	**0.891**
ACC	0.850	**0.873**
MCC	0.702	**0.746**
AUC	0.929	**0.944**

6. Features Description

AAC (Amino Acid Composition)

ACC describes the ratio of each amino acid type in a protein sequence. The ratios of 20 natural amino acids are calculated as:

$$f(r) = \frac{N_r}{N} \quad r = 1, 2, \ldots, 20$$

N_r is the number of the amino acid type r and N is the length of the protein sequence.

DPC (Dipeptide Composition)

DPC refers to a total of 400 dipeptide frequencies in the protein sequence, reflecting the association of adjacent amino acids in the protein sequence. Defined as:

$$f(r, s) = \frac{N_{rs}}{N - 1} \quad r, s = 1, 2, \ldots, 20$$

N_{rs} is the number of dipeptides represented by amino acid type r and type s.

CTD (Composition/Transition/Distribution)

CTD features represent the structural or physicochemical distribution patterns of amino acids in protein sequences[1]. Amino acids can be divided into three groups according to their properties: polar, neutral, and hydrophobic, and each amino acid is coded according to the class to which it belongs. Composition in the CTD is the overall compositional percentage of polar, neutral, and hydrophobic residues in the protein. Transition indicates the frequency in transition, like a transition from class polar to

neutral is the percent frequency with which polar is followed by neutral or neutral is followed by polar in the encoded sequences. Distribution descriptors are the percentage of positions of the first, 25%, 50%, 75%, and 100% residues in the entire sequence in a certain class of codes.

QSO (Quasi-sequence-order)

The quasi-sequence-order descriptors are proposed by Chou[2]. They are derived from the distance matrix between the 20 amino acids.

For each amino acid type, define a quasi-sequence order descriptor as:

$$X_r = \frac{f_r}{\sum_{r=1}^{20} f_r + w \sum_{d=1}^{\max lag} \tau_d} \quad r = 1, 2, ..., 20$$

f_r is the normalized occurrence for amino acid type i and w is the weighting factor (w=0.1). There are the first 20 quasi-sequence-order descriptors. The other 30 quasi-sequence-order descriptors are defined as:

$$X_d = \frac{w\tau_{d-20}}{\sum_{r=1}^{20} f_r + w \sum_{d=1}^{\max lag} \tau_d} \quad d = 21, 22, ..., 20 + \max lag$$

PAAC (Pseudo-amino acid composition)

PAAC was used to describe residues correlation based on their physicochemical properties[3]. The descriptor uses the $20+\lambda$ dimensional vectors to represent the protein sequence. The 20 and λ dimensions denote the amino acid composition and sequence correlation factor.

For any protein X, its pseAAC can be represented as:

$$X = [A_1, A_2, A_3, ..., A_{20}, A_{20+1}, ..., A_{20+\lambda}]^T$$

$20+\lambda$ dimension elements can be formulated as:

$$X_c = \frac{fc}{\sum_{r=1}^{20} f_r + \omega \sum_{j=1}^{\lambda} \theta_j} \quad (1 < c < 20)$$

$$X_c = \frac{\omega \theta_{c-20}}{\sum_{r=1}^{20} f_r + \omega \sum_{j=1}^{\lambda} \theta_j} \quad (21 < c < 20 + \lambda)$$

X_c and ω denote the feature vector and weight factor. Here we set ω as 0.05 as suggested by Chou[3]. τ_k respresents the k-tire sequence correlation factor, it can be formulated as:

$$\tau_k = \frac{1}{L-k} \sum_{i=1}^{L-k} J_{i,i+k} \quad (k < L)$$

$$J_{i,i+k} = \frac{1}{3}\{[H_1(R_i) - H_1(R_{i+k})]^2 + [H_2(R_i) - H_2(R_{i+k})]^2 + [M(R_i) - M(R_{i+k})]^2\}$$

$H_1(R_i)$ is the hydrophobicity value, $H_2(R_i)$ is the hydrophilicity value, and $M(R_i)$ is the side chain mass of the amino acid residue R_i.

APAAC (Amphiphilic Pseudo-Amino Acid Composition)

APAAC incorporates a partial sequence-order effect to the amino acids based on hydrophobicity and hydrophilicity[4]. According to APAAC, a protein is represented as follows:

$$P = [A_1, A_2, A_3, \ldots, A_{20}, A_{20+1}, \ldots, A_{20+\lambda}, \ldots, A_{20+2\lambda}]^T$$

The elements of the first 20 dimensions represent AAC, and the remaining dimensions represent serial correlation coefficients similar to PAAC.

7. BiLSTM structure chart

BiLSTM algorithm was used to obtain further dependency information between protein sequence contexts. The structure of BiLSTM is shown in Supplementary Figure S2.

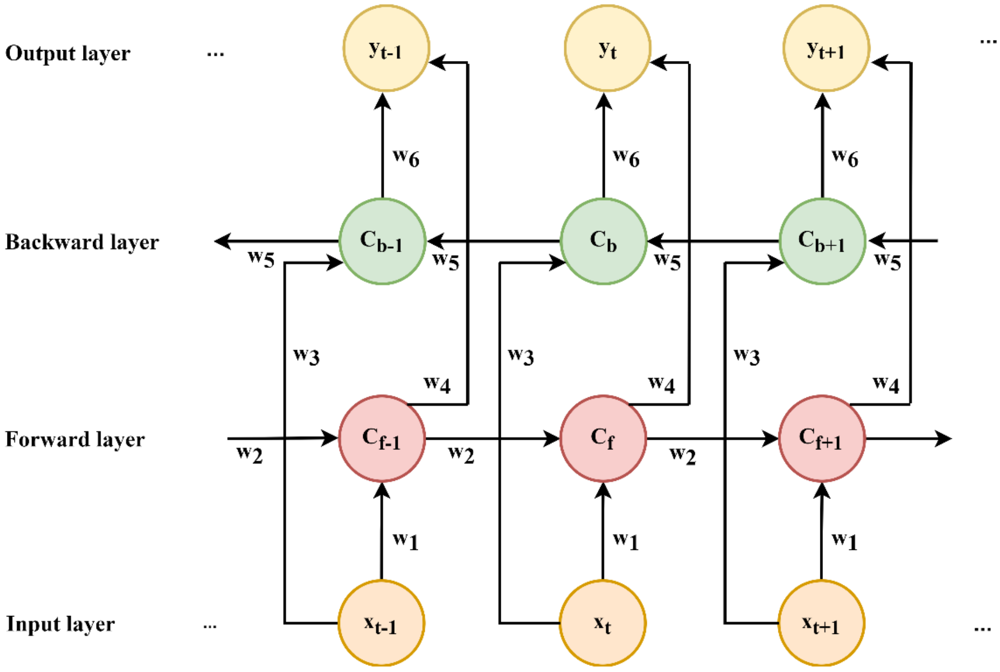

Supplementary Figure S3. BiLSTM structure chart

8. Parameter Settings

The experimental language is Python3.6, the deep learning framework Tensorflow2.4.0 is used to build the experimental model, and the NVIDIA GeForce GTX 960 GPU is used as the computing unit for model training. The model parameter settings are detailed in Supplementary Table S7.

Supplementary Table S7 Model parameters

**Parameters**	**Weights**
max_sequence_length	1500
bio_length	205
input_dim	21
output_dim	128
hidden_size	64
nums_layers	3

epochs	100
batch_size	128
learning_rate	0.001

Max_sequence_length is the length of the input sequence; bio_length is the length of the input biometrics; input_dim and output_dim are the sizes of the input vocabulary of the embedding layer and the dimension of the output word vector respectively; hidden_size is the number of hidden nodes in the BiLSTM layer; nums_layers is the CNN in the DeepTPs model The number of layers; epochs are the number of iterations in the model training process, and to avoid overfitting, early stopping is used.

9. Encoding grouping

According to the physical and chemical properties of amino acid residues, they are divided into 6 groups. For detail, see Supplementary Table S8.

Supplementary Table S8. Classification and coding of amino acid physicochemical properties

**Physical and chemical properties**	**Categories**	**Encode**
Hydrophobic	V, I, L, F, M, W, Y, C	1
Negatively charged	D, E	2
Positively charged	R, K, H	3
Conformational	G, P	4
Polarity	N, Q, S	5
Other properties	A, T	6

References

[1] I. Dubchak, I. Muchnik, C. Mayor, I. Dralyuk, and S. H. Kim, "Recognition of a protein fold in the context of the SCOP classification," Proteins: structure, function, and bioinformatics, vol. 35, pp. 401-407, 1999.

[2] K. C. Chou, "Prediction of protein subcellular locations by incorporating quasi-sequence-order effect."

[3] K. C. Chou, "Prediction of protein cellular attributes using pseudo-amino acid composition," Proteins: Structure, Function, and Bioinformatics, vol. 43, pp. 246-255, 2001.

[4] K.-C. Chou, "Using amphiphilic pseudo amino acid composition to predict enzyme subfamily classes," Bioinformatics, vol. 21, pp. 10-19, 2004.
